# Heavy metal exposures in aerodigestive clinic cohort of infants with reflux or dysphagia

**DOI:** 10.1038/s41598-025-98768-5

**Published:** 2025-04-23

**Authors:** Nan Du, Maritha Du, Tracy Punshon, Rachel Rosen

**Affiliations:** 1 Division of Gastroenterology, Hepatology and Nutrition, Boston Children’s Hospital, Boston, MA USA; 2 Department of Biological Sciences, Dartmouth College, Hanover, NH USA

**Keywords:** Arsenic, Molybdenum, Toxic elements, Reflux, Dysphagia, Gastrointestinal diseases, Paediatric research

## Abstract

Infant cereals (rice/oatmeal), purees, and anti-reflux formulas are often first line treatments for thickening in infants with reflux and oropharyngeal dysphagia. However, there has been growing concern about heavy metal contamination, especially arsenic, in these commonly used thickeners. This is a particular concern in infants who may be more susceptible to heavy metal neurotoxicity. The study aimed to assess whether there are differences in heavy metal levels, particularly arsenic, in infants with reflux or oropharyngeal dysphagia while on different thickeners. We performed a single center study in infants (< 1 year of age) with diagnosis of reflux or oropharyngeal dysphagia who were seen between December 2021–2023. Participants submitted urine samples and completed dietary questionnaires to assess their elemental exposures. The primary outcome of interest was urinary arsenic concentrations, though other elements were also measured. Of the 56 infants, 27 were on gelmix^®^ or purees, 19 were on Enfamil AR and 10 were on infant oatmeal/rice cereal as a thickener. The median total urinary arsenic concentration did not differ between groups (*p* = 0.086) and levels between groups were well below the Agency for Toxic Substances and Disease Registry (ATSDR)’s toxicity limits. Infants with higher number of servings of alternative arsenic sources via their solid food were more likely to have higher urinary arsenic level (*p* = 0.001), suggesting a potential need for the FDA to implement stricter food supply regulations. Only molybdenum had significant difference in levels between thickeners (*p* = 0.0012). Even in high-risk patients, urinary arsenic concentrations did not differ between thickener groups.

## Introduction

Thickened feeding is a commonly employed approach in pediatric clinical practice to effectively treat both gastroesophageal reflux and oropharyngeal dysphagia in infants and young children^1^. Various options are available for thickening including infant rice cereal, oatmeal, purees and anti-reflux formula. However, a recent report by United States House Committee has raised concerns about the presence of metals in baby foods and rice cereal sold in the United States, two of the commonly used thickeners^2,3^. Most of the concern has come from direct testing of baby food products from Consumer Reports, which has shown varying higher concentration of assorted heavy metals in baby and toddler foods^4,5^.

Heavy metals are defined as toxic elements, such as arsenic, lead, mercury and cadmium, all of which occur naturally in the environment and can be elevated by prior industrial and agricultural activities^5^. The United States Food and Drug Administration (FDA) and World Health Organization have both declared that arsenic is dangerous to human health, especially for children, who are at highest risk of neurotoxic effects given that toxic elements naturally bioaccumulate over time^6,7^. In particular, chronic arsenic exposure has been associated with neurological and cognitive dysfunction, defects in humoral immunity, increased risk of respiratory illness, metabolic disorders and impaired growth^8–12^. Early and chronic exposure to other heavy metals such as lead, mercury and cadmium have also been associated with developmental delay and inhibited growth^13–15^.

There have been multiple voluntary recalls by the FDA pertaining to questionable levels of heavy metals in rice cereal, applesauce, chocolate and sweet potato snacks^16,17^. While the United States FDA have recently proposed maximum limits for the amount of lead, cadmium and arsenic in foods^18^, these limits are solely recommendations rather than mandatory rules in part due to a lack of monitoring infrastructure and a lack of an oversight body to enforce exceeding such limits^18^.

Despite these contamination concerns, there are limited data on the actual exposure to toxic elements by infants under one year of age, particularly in a population that requires the use of thickeners (which have been noted to contain potentially elevated levels of toxic elements)^19^. The prior pilot work conducted by the New Hampshire Birth Cohort study (NHBCS), a longitudinal study investigating the role of contaminants in environment effect on infants and pregnant women, showed some elemental exposure, but their study population only included healthy infants at 6 weeks of age (which is likely before any infants would have started thickening)^3^. Thus, there is a gap in the literature on heavy metal exposure in infants with medical indication for thickening. Our primary goal of this study was to assess whether there are differences in arsenic and other heavy metal levels directly measured in infants with reflux or oropharyngeal dysphagia while on different thickeners.

## Methods

### Study design

**Fig. 1:**
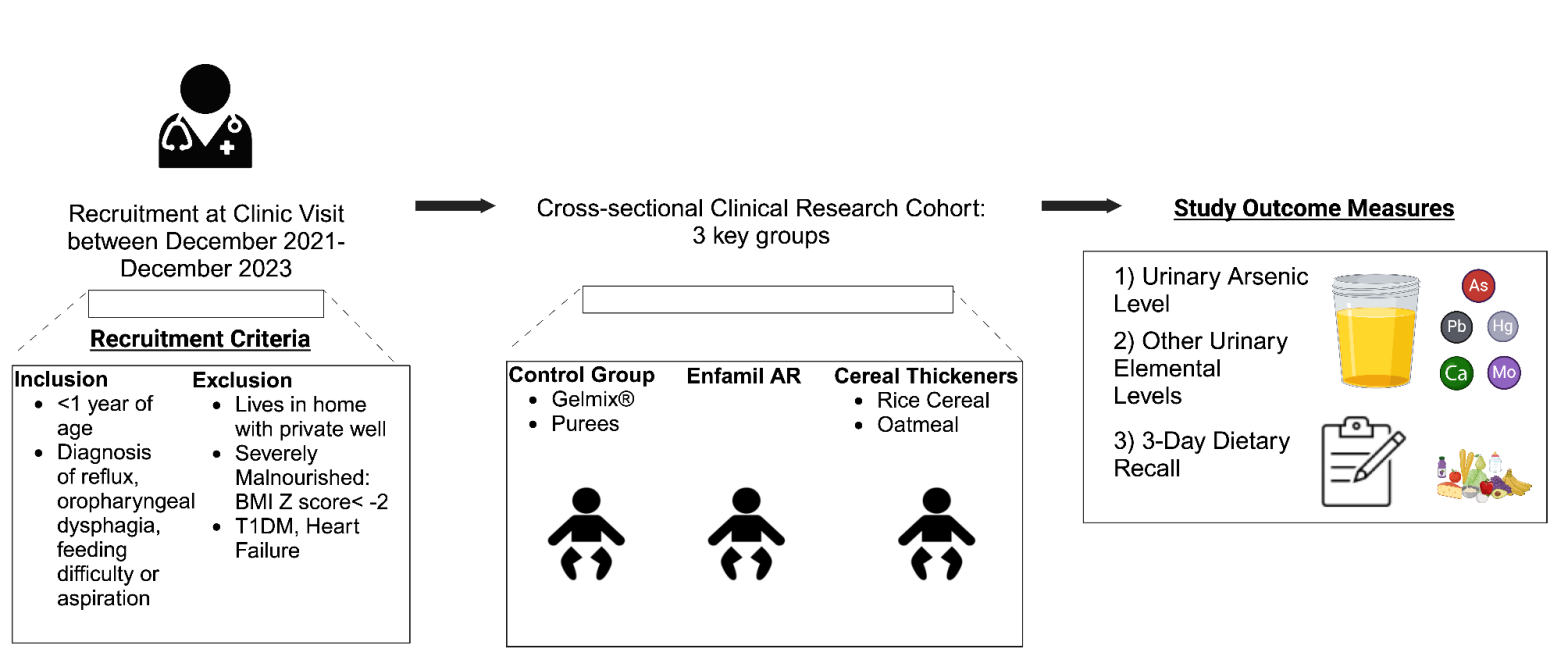
Study schematic. BMI is defined as body mass index. T1DM is defined as Type 1 Diabetes Mellitus. As is defined as arsenic. Pb is defined as lead. Hg is defined as mercury. Ca is defined as calcium. Mo is defined as molybdenum.

We performed a single center study of infants (< 1 year of age) with diagnosis of reflux, oropharyngeal dysphagia or aspiration who were seen between December 2021 to December 2023. Infants with type 1 diabetes, severe malnutrition, or who live in a home with private well-water were excluded (Fig. 1). Participants submitted single and, when possible, serial urine samples with an accompanying 3-day dietary questionnaire to assess dietary heavy metal exposures. The primary outcome of interest was urinary arsenic level when infants were on different types of thickeners. Infants on gelmix^®^ or purees (i.e., patients without grains) were considered the control group. We also compared the median level of 21 other nonessential and essential element measurements in urine while infants were on different types of thickeners. We also collected other data including reflux symptom severity index, puree exposure, current age of infant, socioeconomic status, infant’s sex, and whether there was any household tobacco smoke exposure (given that tobacco and its smoke contains arsenic).

### Urinary arsenic biomarker and urine collection

The primary outcome was the measured urinary arsenic level. Urine is the most reliable, stable, and accurate measurement of short term (e.g., three day) arsenic exposure and it can also be speciated as inorganic arsenic and its metabolites, which allows for all biologically relevant forms of arsenic to be assessed^11,20^. Urine is the main route of excretion of most arsenic species with half-life of 4 days and is the most commonly used standard biomarker for epidemiological studies on arsenic exposure^21^. Spot urine samples have been demonstrated as adequate and cost effective biomarkers for measured arsenic exposure when compared to 24 h urine collection samples^22^. Spot urine samples were collected on the third day of the food diary using cotton pads that were placed in the infant diaper, following protocol from Carignan et al. and Kargas et al.^23,24^. If the pads were soiled by feces, mothers were instructed to discard the pads and make another attempt at collection. The saturated pads were placed in a collection cup and stored in a cooler with frozen ice packs. Within 24 h, the urine-soaked cotton balls were placed in a syringe, and urine was extracted from the cotton pads via plunger depression. Samples were aliquoted into 1.8 ml vials within 24–72 h and frozen at -80 C until analysis^24^. As noted by prior studies, there is limited benefit from urine adjustment(e.g., creatinine adjustment method) as there is a strong correlation between adjusted and non-adjusted urine samples with no significant difference in the urinary arsenic concentration^25–27^.

### Laboratory analysis

Urinary arsenic, as well as 21 other essential and nonessential elements (Be, V, Cr, Mn, Fe, Co, Ni, Cu, Zn, Se, Sr, Mo, Ag, Cd, Sn, Sb, Ba, Hg, Ti, Pb, and U), were measured at the Trace Element Analysis (TEA) Core at Dartmouth College. Previous studies have established that dietary intake is reflected by urinary concentrations, and thus these urine levels would be a useful biomarker for several of the heavy metals (cadmium, mercury, nickel, uranium, cobalt, molybdenum, and selenium)^28–30^. Elemental analysis was conducted using inductively coupled plasma mass spectrometry (Agilent 8900, Agilent Technologies Headquarters, Santa Clara, CA, USA), which is the reference standard for urine heavy metal analysis^31^. Urinary elemental species concentrations were determined using the Agilent 8900 ICP-MS interfaced with an Agilent liquid chromatograph 1260 equipped with Thermo AS7, 2 × 250 mm column and a Thermo AG7, 2 × 50 guard column^32^. Reference materials are run after each calibration and every 20 samples. Reference samples used for urine are Seronorm Level 1 and 2 urine and UTAK laboratory quality certified urine standards. The estimated total arsenic was defined as sum of arsenite, arsenate, monomethylarsonic acid and dimethylarsinic acid. Arsenobetaines were not included given these are considered nontoxic forms of organic arsenic and tend to be excreted without being metabolized^33^.

### Dietary recall

Infants’ parents or caregivers were asked to complete a food diary at the end of each day. The food diary included details of infants’ food and beverage intake during 3 consecutive days (e.g., type of water used (tap, bottled, or spring water), time of feeding and type and amount of foods/beverage consumed); this dietary measurement is more accurate and less likely to overestimate infant energy and nutrient intake compared to a single 24-hour dietary recall^34^. Families were specifically asked about foods that may contain arsenic and toxic elements divided in the following categories (with examples): fruit and juice (apple, pear and grapes), meat and fish (canned tuna fish, dark meat fish, other fish, chicken), prepared infant and toddler meals (Gerber™ graduates, Earth’s Best Organic), snack foods (puffs, rice cakes, granola bars, snack bars), cold or hot cereals and other rice and rice-based products. We also calculated the cereal load per ounce of formula for those who were consuming oatmeal and rice cereal thickener. We quantified total rice servings over the 3-day period, as well as servings of alternative sources of dietary arsenic (i.e. not from the thickener or rice containing products).

### Statistical analysis

The data were analyzed with R statistical software version 2023.03.0 + 386. Standard descriptive statistics (e.g. means and standard deviations for continuous variables and proportions for categorial variables) were used to summarize data. We compared urinary elemental concentrations (expressed as parts per billion/million) between thickener groups using a Kruskal Wallis Test and performed multivariate regression analyses to determine predictors of urine heavy metal levels. For patients with two separate urine samples, we considered them independent measurements. P values of < 0.05 were considered significant unless otherwise indicated. The Boston Children’s Hospital IRB approved the study protocol.

### Conference presentation

Preliminary data was presented as an abstract/poster at North American Society for Pediatric Gastroenterology Conference in 2023.

## Results

**Fig. 2:**
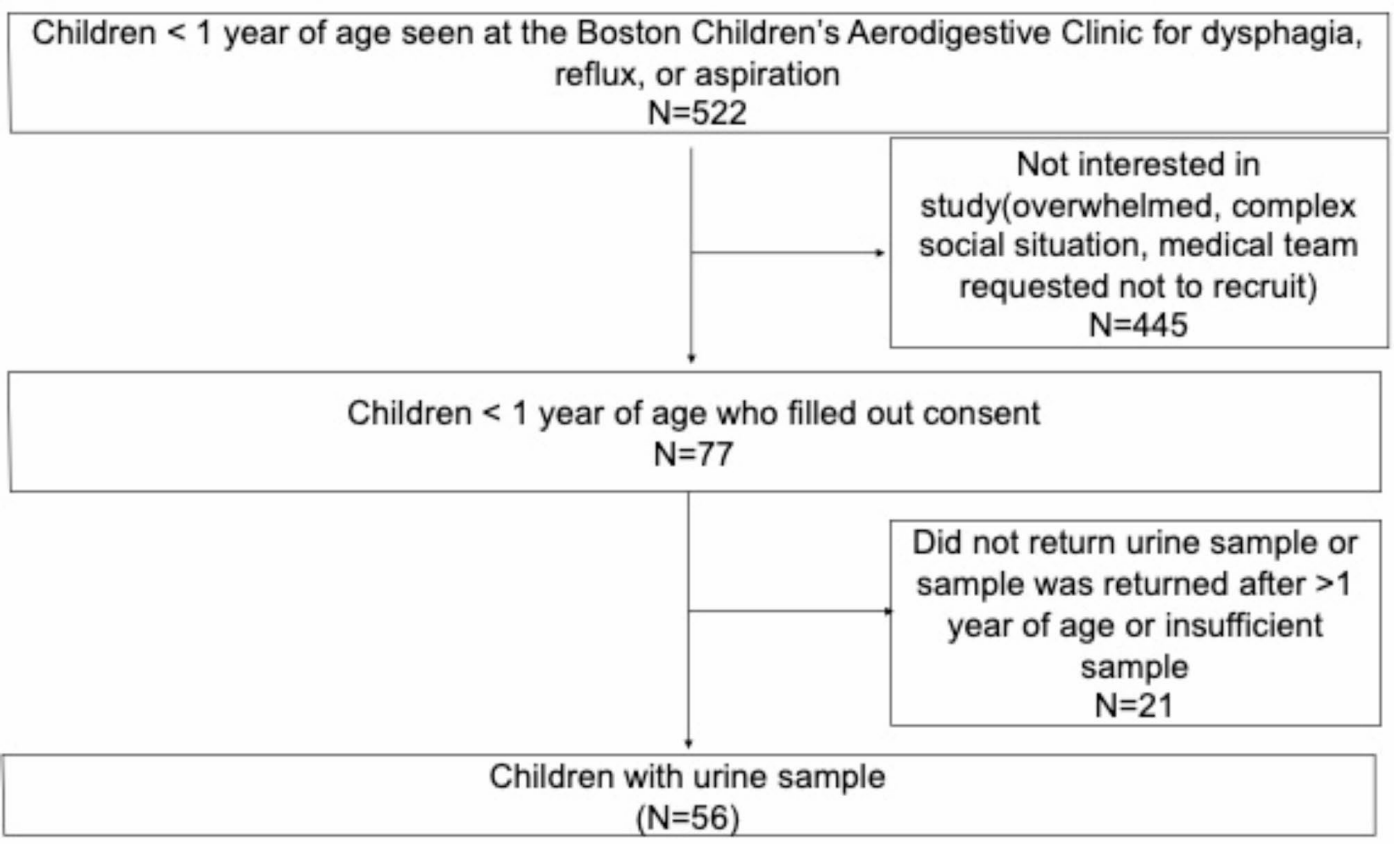
Recruitment schematic.

We approached a total of 522 infants with dysphagia, reflux or aspiration who were seen at a single medical center. Four hundred and forty-five participants were not interested in the study with reasons including being overwhelmed, having a complex social situation, inability to wait for wet diaper, or inability to wait to complete questionnaire or using well water or medical team requesting that the participant not be recruited at that time. Of the 77 who consented to participate in the study, 56 (73%) infants provided one sample of urine (Fig. 2). 57% of the infants who provided one sample of urine were female with a mean age of 7.0 months (± 2.8 months) old at the time of sample collection (Table 1). Furthermore, 95% of the participants self-identified as white, 76% (43/56) were from a married household, 64% (36/56) of infants were from a household where the annual income was > $100,000 and 84% (47/56) of infants had at least one parent that had a college degree. All infants were from non-smoking households.

**Table 1:** Demographics and clinical characteristics of recruited infants with urine sample.

	*n*	%
*Infant clinical characteristics*		
Age at sample (months)	7	
Female	32	57.14%
White race	53	94.64%
Puree/solid exposure	36	64.29%
Thickening reason		
GERD	8	14.29%
Oropharyngeal dysphagia	27	48.21%
Both	21	37.50%
Neither	0	0.00%
Thickener		
Gelmix^®^/purees(control)	27	48.21%
Enfamil AR	19	33.93%
Oatmeal/rice cereal	10	17.86%
IDDSI and levels of thickness		
Puree	20	35.71%
Level 1	20	35.71%
Level 2	4	7.14%
Level 3	12	21.43%
Average tsp/ounce in oatmeal/rice(STD)	1.63	0.85
*Household demographics*		
Married martial status	43	76.79%
Head of household education level		
Some HS-high school diploma	1	1.79%
Some college	6	10.71%
Undergraduate degree	16	28.57%
Graduate	30	53.57%
Missing	3	5.36%
Head of household employed, working > 40 h	48	85.71%
Combined household income		
<$60,000	7	12.50%
$60,001-$80,000	5	8.93%
$80,001-$100,000	3	5.36%
More than $100,0000	36	64.29%
Prefer not to answer	5	8.93%

The majority (64%) of the infants had already been exposed to purees (i.e. complementary foods) at the time of urine collection. In general, parents of the infants (59%) used bottled water when preparing formula or food items that required water, while 29% of the participants used tap water. 57% had consumed at least one serving of a food that may be considered alternative source of dietary arsenic, such as applesauce, brussels sprouts, carrots, or sweet potato puree. 32% (18/56) had consumed at least one serving of rice-containing food (rice cereal, brown rice syrup in ingredients). For the patients who had seen a speech language pathologist for thickening recommendations, 35% were taking IDDSI (International Dysphagia Diet Standardization Initiative) level of slightly thick, 7% were taking IDDSI level of mildly thick, 21% were moderately thick, and 35% were on IDDSI level of pureed food. In individuals with oatmeal/rice cereal thickening, the average thickening amount is 1.63 (*±* 0.85) teaspoon/ounce with target IDDSI level aim of 2.35 (*±* 0.86) or mildly thick.

**Fig. 3:**
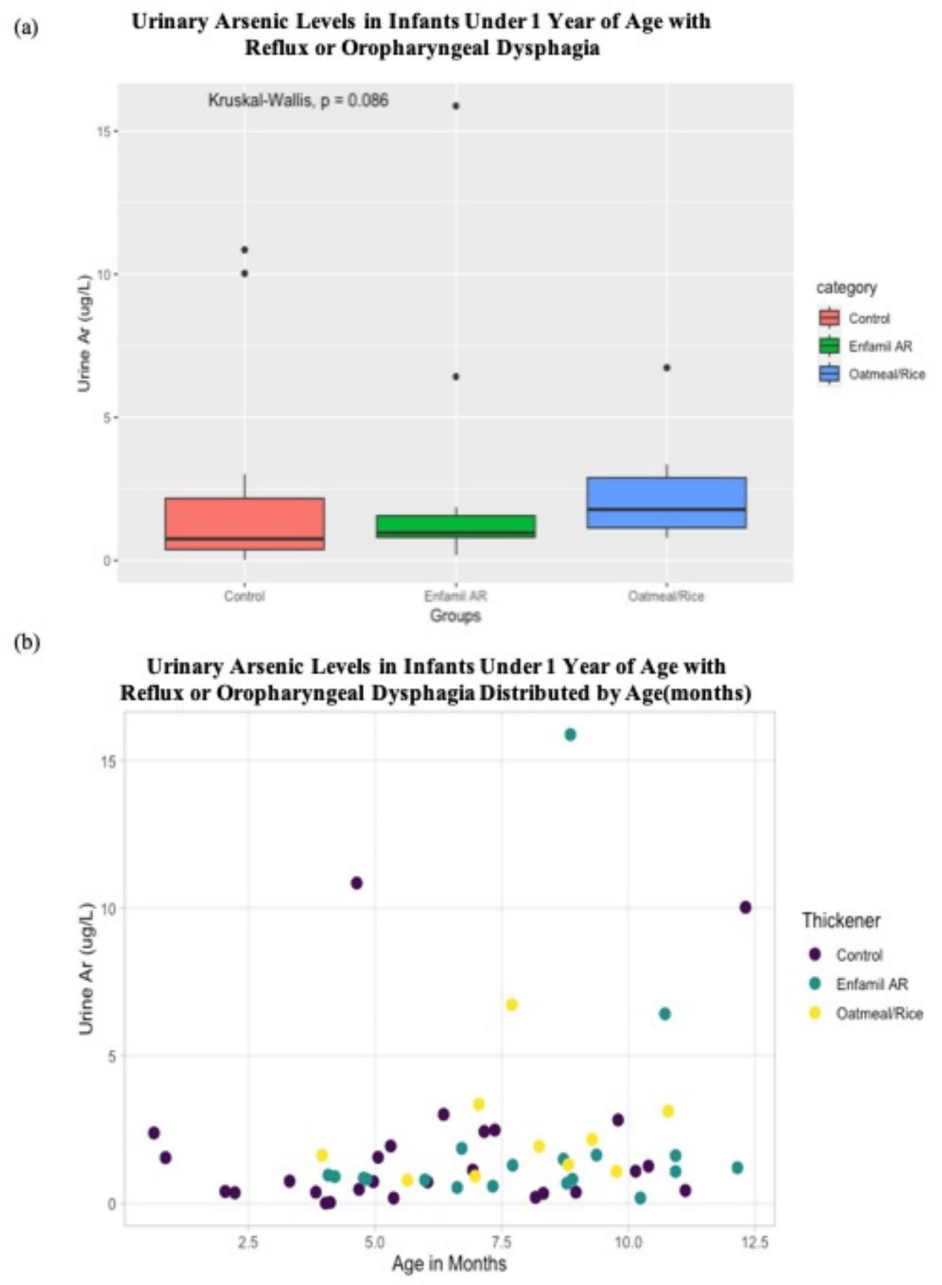
Urinary arsenic levels in infants under 1 year of age with reflux or oropharyngeal dysphagia. (**a**) Urine samples are separated. by thickening category (purees/control, Enfamil AR and oatmeal/rice) (**b**) Urine samples are distributed by age of infant and type of thickener (purees/control, Enfamil AR and oatmeal/rice).

Of the 56 infants, 27 were on gelmix^®^ or purees (controls), 19 were on Enfamil AR and 10 were on infant oatmeal/rice cereal as a thickener. Eleven infants (included in the control group) were breastfed with 4 of those supplementing with formula. Infants in the control group tended to be male and slightly younger in age. Otherwise, there was no difference in race, parental marital status, parental education level or household income amongst the three groups. The median total urinary arsenic concentration did not differ between groups (Fig. 3a, *p* = 0.086) (1.78 µg/L, 0.96 µg/L, 0.76 µg/L for respectively oatmeal/rice cereal, Enfamil AR and Control groups). There was a trend towards more arsenic in the urine as children aged but was not significant (Fig. 3b, *p* = 0.09). All participants had a urinary arsenic level significantly less than 100 µg/L, the toxic level held by Agency for Toxic Substances and Disease Registry (ATSDR) in the US Department of Health and Human Services^35^. We performed a multivariate analysis including a model adjusting for age, puree exposure, number of servings of alternative sources of dietary arsenic, servings of rice, and thickener status to predict urinary arsenic exposure; only alternative arsenic dietary servings predicted urinary arsenic exposure (*p* = 0.0012).

Additionally, analysis of 21 other elements revealed statistically significant differences in concentrations for 9 of the elements (beryllium, manganese, iron, molybdenum, cadmium, tin, antimony, mercury, and lead) (Table 2). None of the concentrations of elements in urine were significantly above the toxic levels set by the ATSDR in the United States Department of Health and Human Services^36^ ATSDR typically derives these screening levels based on existing toxicological studies and epidemiological data in adults; however, we did not independently validate whether these ATSDR levels are sufficiently protective of infants.

**Table 2:** Essential and non-essential element concentrations (µg/L) in urine samples for refluxing/aspirating infants < 1 year of age.

Heavy metals	Control			Enfamil AR			Oatmeal/rice thickener			*p* value
	Median	Range	Q1, Q3	Median	Range	Q1, Q3	Median	Range	Q1,Q3	
Arsenic	0.74	[0.0, 10.9]	[0.4, 2.2]	0.96	[0.2, 15.9]	[0.81, 1.56]	1.78	[0.8, 6.7]	[1.14, 2.89]	0.086
Beryllium	0	[0.0, 0.5]	[0.00, 0.10]	0	[0.0, 0.09]	[0.00, 0.00]	0.04	[0.0,0.3]	[0.00, 0.11]	0.028
Vanadium	0	[0.0, 1.5]	[0.00, 0.5]	0	[0.0, 0.6]	[0.00, 0.00]	0.25	[0.0, 1.0]	[0.06, 0.44]	0.069
Chromium	0.37	[0.0, 3.4]	[0.00. 0.7]	0.72	[0.0, 7.9]	[0.10, 0.94]	0	[0.0, 3.1]	[0.00, 1.93]	0.53
Manganese	37.79	[4.0, 173]	[15.9, 80.1]	8.45	[0.05, 98.5]	[4.53, 32.28]	34.56	[3.8, 127]	[24.31, 63.33]	0.010
Iron	39	[7.9, 330.5]	[18.6, 65.9]	30.38	[2.4, 757.9]	[15.94, 47.82]	67.95	[24.8, 186]	[49.8, 99.75]	0.035
Cobalt	0.13	[0.07, 0.5]	[0.1, 0.23]	0.15	[0.02, 6.4]	[0.10, 0.25]	0.26	[0.06, 4.4]	[0.13, 0.37]	0.30
Nickel	2.05	[0.60, 18.3]	[1.3, 2.59]	1.55	[0.43, 16.2]	[1.41, 3.49]	4.76	[1.3, 21.9]	[1.65, 12.11]	0.15
Copper	13.98	[0.00, 67.5]	[8.5, 20.8]	11.89	[0.0, 122.3]	[9.75, 21.30]	20.89	[5.5, 73.3]	[10.53, 28.59]	0.55
Zinc	160.65	[87.5, 13442]	[113.2, 1097.6]	513.11	[69.59, 66601]	[285.65, 968.6]	2947.5	[52, 34750]	[328.7, 9456]	0.24
Selenium	22.35	[4.0, 140.7]	[13.2, 41.0]	41.1	[6.1, 83.7]	[23.60, 49.65]	39.74	[4.0, 113.3]	[23.28, 64.29]	0.15
Strontium	91.69	[30, 434]	[68.3, 125.1]	70.95	[9.7, 540.5]	[35.92, 119.48]	58.46	[19.9, 216.0]	[32.85, 82.97]	0.16
Molybdenum	14.76	[0.00, 276.14]	[3.9, 25.7]	19.12	[2.5, 151.7]	[14.15, 49.41]	100.56	[9.8, 678.3]	[40.01, 134.04]	0.0012***
Silver	0.001	[0.00, 0.3]	[0.00, 0.008]	0.004	[0.0, 0.06]	[0.00, 0.0093]	0	[0.0, 0.09]	[0.00, 0.013]	0.99
Cadmium	0.069	[0.03, 0.5]	[0.05, 0.09]	0.071	[0.01, 0.2]	[0.045, 0.106]	0.16	[0.06, 0.70]	[0.08, 0.23]	0.025
Tin	0.61	[0.2, 8.2]	[0.44, 1.2]	0.51	[0.00, 1.5]	[0.32, 0.84]	1.2293	[0.6, 4.4]	[0.93, 2.32]	0.0046
Antimony	0.2	[0.04, 1.6]	[0.12, 0.3]	0.15	[0.02, 14.4]	[0.09, 0.59]	0.68	[0.15, 54.5]	[0.32, 1.05]	0.022
Barium	13.27	[1.7, 439.4]	[6.7, 29.3]	6.54	[0.60, 39400]	[3.64, 13.42]	15.1	[1.25, 37.4]	[8.65, 25.51]	0.13
Titanium	0.2	[0.00, 2.9]	[0.03, 0.3]	0.12	[0.00, 2.1]	[0.03, 0.27]	0.35	[0.0, 0.8]	[0.11, 0.49]	0.34
Mercury	0.031	[0.00, 0.80]	[0.00, 0.1]	0	[0.00, 0.3]	[0.00, 0.10]	0.21	[0.0, 2.1]	[0.07, 0.39]	0.025
Lead	0.33	[0.00, 3.6]	[0.2, 0.6]	0.20	[0.00, 1.8]	[0.04, 0.32]	0.39	[0.2, 2.8]	[0.31, 1.14]	0.031
Uranium	0.59	[0.04, 7.3]	[0.23, 1.09	0.30	[0.00, 2.0]	[0.16, 0.57]	0.7	[0.05, 2.5]	[0.61, 1.31]	0.078

The reported values refer to median (range, interquartile range: Q1-Q3). The As concentrations refer to the sum of inorganic arsenic, monomethylarsonic acid, and dimethylarsinic acid.

Only molybdenum, tin, cadmium, mercury, and uranium have had literature that supports urine as a suitable biomarker for dietary exposure^30,37^. Using a conservative Bonferroni correction to adjust for multiple comparisons, only molybdenum showed significant difference in levels between thickening groups (*p* = 0.0012, as *p* < 0.0022 would be considered significant). Concentrations of all of the elements except molybdenum fell within ranges previously measured in the NHBCS^3,37^. When performing a multivariate analysis including a model adjusting for age, puree exposure, alternative arsenic dietary sources, and category of thickener, oatmeal/rice cereal thickener was associated with higher levels of urinary molybdenum (*p* = 0.00198).

High element exposure was largely seen in four infants (two from control group, one on Enfamil AR and one on oatmeal/rice cereal thickener) who collectively had the highest levels of 16 of the 21 elements measured with each having elevated levels for 2–5 of the elements. There was no defining demographic characteristic that united these four infants.

## Discussion

The study is the first to examine a vulnerable population, early infants (aged 1–12 months) with reflux/oropharyngeal dysphagia, whose medical treatment may place them at a hypothetical increased risk for toxic element exposure due to use of thickeners and purees. Our study is one of the first to report on data gathered after the FDA’s action level on arsenic was finalized in August 2020^38^ and may be the most accurate representation of current food exposures for infants in the United States. While prior studies have demonstrated dietary exposure to essential and nonessential elements via human milk, formula, as well as solid exposure (especially rice cereal), these have been limited to healthy infants and samples that were collected prior to implementation of FDA’s action level^37,39^. Our cross-sectional study found no significant difference in urinary excretion of heavy metals, except for molybdenum, among young infants less than 1 year of age consuming different thickening agents.

### Arsenic

Safety concerns have previously been raised because of elevated levels of inorganic arsenic in all forms of rice including infant cereals, brown rice syrup and certain solid food purees. Arsenic exposure has been linked to neurotoxicity and long-term cancer risk in areas with significant environmental arsenic contamination with urinary arsenic levels of > 400 µg/L^40^. However, this level of contamination is not common in the United States or Europe. The urinary arsenic levels for our observed cohort were within range of an earlier study performed on healthy infants under 1 year of age who had been recruited by the NHBCS^19^. In small subsets of the NHBCS infants, feeding mode and increased rice servings were predictive of higher urinary arsenic levels^3,19,39^. We did not identify any significant differences in the amount of urinary arsenic between children on different thickeners, and urinary arsenic concentrations were well below the ATSDR-published safe levels. Of note, the ATSDR does not provide any scientific basis or citations to literature for their set level, so we are unable to determine if 100 µg/L is sufficiently protective for infants. Many of the studies, which have been mostly performed in adults, seem to suggest increased health risks are associated with urinary arsenic levels above 50 µg/l^41,42^. In the limited epidemiologic reports conducted in children with elevated arsenic exposure, moderate exposure to arsenic (defined as > 50 µg/L) may have adverse effect on cognition and development^43^.

Our study did not find a correlation between the urinary arsenic concentration and number of rice/rice flour servings that the infant consumed. We had similar percentage of infants who had consumed foods made with rice or sweetened with rice syrup in the past 3 days (32% vs. 24% respectively for our cohort in comparison to prior work) as well as similar rice servings per day (median of 2 servings over 3 days compared to 5–6 servings over week)^23^. This may reflect a reduction in food contamination in the face of growing publicity over arsenic in cereals placing pressure on industry to independently decrease their levels^16,17,39^. We did see an increase in arsenic levels with more puree intake, suggesting that arsenic levels in the urine are complex and that banning or avoiding one food type may not address the more widespread contamination throughout the food chain, an issue which has been raised by a later congressional report^18^. However, given the overall low levels of arsenic contaminants in the various thickening agents, parents and medical providers should feel more reassured that thickeners (via formula, purees or cereal) do not significantly increase infants’ urinary arsenic concentration.

### Molybdenum

Molybdenum (Mo) is essential for normal growth and development, and is commonly used as a fertilizer for various crops. Currently, the main source of molybdenum exposure is through oral consumption of legumes vegetables, milk (breastmilk and formula) and animal organs^44^. Given that urine is the main elimination route for Mo, urinary Mo level is considered a useful biomarker of exposure and is sensitive to changes in dietary Mo intake^28,45^. In our study, urinary Mo was significantly higher in those patients who received thickening with oatmeal and rice cereal, which is likely due to grain products being the primary dietary source of molybdenum^44^. The mean urinary Mo levels in our study was 51.8 µg/L which was similar to the mean of 51.6 µg/L in the United States (derived from 486 individuals between 3 and 5 years of age who participated in the National Health and Nutrition Examination Survey during 2015–2016)^46,47^. Furthermore, all measured values were below the Institute of Medicine’s biomonitoring equivalent for urine molybdenum (200–7500 µg/L), which is a level set to protect against toxicity^48^. There is limited data on human toxicity of Mo given that it is rapidly excreted in urine, with skeletal abnormalities observed in rats with high Mo toxicity^49^. There was one case report noting gout-like syndrome and pneumoconiosis with excessive persistent ingestion of molybdenum^50^. A prospective cohort study based in Mexico also identified an association between elevated prenatal Mo exposure and lower psychomotor development scores^51^. Overall, Mo toxicity is rare, and the higher levels in infants with thickening may reflect the kidney’s appropriately excreting Mo rather than persistent chronic exposure.

There are several limitations to our study. First, the majority of patients only provided a single sample so longitudinal assessment over time as the diet expands was not performed. This longitudinal analysis would allow for a better sense of a causal relationship. A second limitation is that this study relied on a self-reported dietary data so we had to extrapolate about possible amounts of toxins in foods rather than testing samples of the subjects’ meals or drinking water. While the dietary information gathered with the food diary may not represent an infant’s typical food pattern, it does provide a concise snapshot. Furthermore, since the diaries were kept in real time, recall bias should be less of a concern. Third, while our study is adequately powered to address our main question on differences in urinary arsenic among the different groups, our study may lack statistical power for additional heavy metal comparisons which may have risk of false negative results. Fourth, our study is limited by a lack of racial and ethnic diversity in our clinic patient population, which may limit the generalizability of our findings. Thus, we may be underestimating heavy metal exposure in infants given some prior studies showing the potential role of social drivers of residential location on individual’s propensity for exposure to metals in the environment^52^.

## Conclusion

In this study, we did not identify any differences in the concentrations of urinary arsenic between children on different thickeners, and urinary arsenic values were well below the ATSDR-published safe levels. However, more longitudinal studies need to be conducted to assess for changes in urinary elements over time. Furthermore, when analysis was expanded to include exposure to potentially other toxic essential and nonessential elements, only molybdenum showed increased levels for oatmeal and rice cereal thickeners, which suggests that future studies may need to include this element for additional analyses. Overall, this study represents the largest cohort of infants following FDA guideline changes and offers some reassurance for pediatricians caring for infants who need thickening for GERD or swallowing difficulties.

## Data Availability

The data that support the findings of this study are not openly available due to reasons of sensitivity and are available from the corresponding author upon reasonable request and with permission from Boston Children’s Hospital.

## Abbreviations

Asi: Inorganic arsenic
Enfamil AR: Enfamil anti-reflux
FDA: Food and Drug Administration
ATSDR: Agency for Toxic Substances and Disease Registry
IDDSI: International Dysphagia Diet Standardization Initiative
Mo: Molybdenum
NHBCS: New Hampshire Birth Cohort Study
